# Kat2a and Kat2b Acetyltransferase Activity Regulates Craniofacial Cartilage and Bone Differentiation in Zebrafish and Mice

**DOI:** 10.3390/jdb6040027

**Published:** 2018-11-12

**Authors:** Rwik Sen, Sofia A. Pezoa, Lomeli Carpio Shull, Laura Hernandez-Lagunas, Lee A. Niswander, Kristin Bruk Artinger

**Affiliations:** 1 Department of Craniofacial Biology, School of Dental Medicine, University of Colorado Anschutz Medical Campus, Aurora, CO 80045, USA; rwik.sen@ucdenver.edu (R.S.); Lomeli.Shull@ucdenver.edu (L.C.S.); ana-laura.Hernandez@ucdenver.edu (L.H.-L.); 2 Cell Biology, Stem Cells, and Development Graduate Program, University of Colorado Anschutz Medical Campus, Aurora, CO 80045, USA; Sofia.pezoa@ucdenver.edu; 3 Department of Pediatrics, University of Colorado Anschutz Medical Campus, Aurora, CO 80045, USA; Lee.Niswander@colorado.edu; 4 Molecular Cellular and Developmental Biology, University of Colorado Boulder, Boulder, CO 80309, USA

**Keywords:** craniofacial skeleton, cranial neural crest cells, histone H3K9 acetylation, GCN5, PCAF

## Abstract

Cranial neural crest cells undergo cellular growth, patterning, and differentiation within the branchial arches to form cartilage and bone, resulting in a precise pattern of skeletal elements forming the craniofacial skeleton. However, it is unclear how cranial neural crest cells are regulated to give rise to the different shapes and sizes of the bone and cartilage. Epigenetic regulators are good candidates to be involved in this regulation, since they can exert both broad as well as precise control on pattern formation. Here, we investigated the role of the histone acetyltransferases Kat2a and Kat2b in craniofacial development using TALEN/CRISPR/Cas9 mutagenesis in zebrafish and the *Kat2a^hat/hat^* (also called *Gcn5*) allele in mice. *kat2a* and *kat2b* are broadly expressed during embryogenesis within the central nervous system and craniofacial region. Single and double *kat2a* and *kat2b* zebrafish mutants have an overall shortening and hypoplastic nature of the cartilage elements and disruption of the posterior ceratobranchial cartilages, likely due to smaller domains of expression of both cartilage- and bone-specific markers, including *sox9a* and *col2a1*, and *runx2a* and *runx2b*, respectively. Similarly, in mice we observe defects in the craniofacial skeleton, including hypoplastic bone and cartilage and altered expression of *Runx2* and cartilage markers (*Sox9*, *Col2a1*). In addition, we determined that following the loss of Kat2a activity, overall histone 3 lysine 9 (H3K9) acetylation, the main epigenetic target of Kat2a/Kat2b, was decreased. These results suggest that Kat2a and Kat2b are required for growth and differentiation of craniofacial cartilage and bone in both zebrafish and mice by regulating H3K9 acetylation.

## 1. Introduction

Craniofacial bones and cartilage of the head and neck must undergo proper differentiation to form specific skeletal elements in adult vertebrates. These skeletal elements are derived from cranial neural crest cells (cNCC), a population of neural crest cells that originate at the rostral region of the embryo and emigrate from the dorsal part of the neural tube during vertebrate embryogenesis [1,2,3,4,5,6,7,8,9,10]. These migratory and multipotent cells migrate to designated locations in the branchial arches and differentiate into either the craniofacial skeletal structures, neurons, glia of the peripheral nervous system, or melanocytes [1,4,5,9,11]. Following their arrival in the branchial arches, cNCC interact with other tissues, including the ectoderm, endoderm, and mesoderm, leading to the formation of the mandibular and maxillary prominences in mammals, and the viscerocranium and neurocranium in zebrafish [9,12,13,14,15].

The neural crest cell lineage is known as the “fourth germ layer” because of the diversity of cell types that these cells give rise to, similar to the other three primary germ layers that form in the developing embryo [16,17]. During evolution, neural crest cells have transformed the chordate body plan to include formation of the cartilage and bone of the craniofacial skeleton, greatly expanding the cell diversity from those observed in more basal chordates. A conserved gene regulatory network for the development of neural crest cells exists in all vertebrates, and has been determined to be similar among the various phyla where it has been examined [16,18]. Neural crest cells and mesodermal cells both contribute to the craniofacial skeleton in all craniates, and the majority of the skull is derived from neural crest cells [9,19,20,21]. Formation of the craniofacial skeleton involves two kinds of ossification: endochondral and intramembranous. This dual process of ossification is conserved in teleost fish, including zebrafish, as well as in mammals [19,22,23,24,25,26,27]. Endochondral ossification occurs from a cartilaginous scaffold of chondrocytes, which is replaced by osteoblasts and bone matrix [19,27]. Some of the craniofacial bones that are generated by this process include the zebrafish neurocranium, branchial arches, ceratohyal, ceratobranchial, and hyomandibula. Intramembranous ossification occurs when mesenchymal stem cell precursors directly differentiate into osteoblasts and form the mineralized bone matrix [19,27,28]. This process generates the zebrafish craniofacial bones, including the opercle, parasphenoid, branchiostegal rays, cleithrum, mandible, maxilla, and dermal plates of the skull.

In mouse, streams of cNCC will migrate from the diencephalon and rostral rhombomeres to populate the first through fourth branchial arches. Here, cNCCs contribute to the maxilla and mandible, the cartilage of the nasal capsule, Meckel’s cartilage, which will contribute to the mandibular symphysis and the malleus and incus, and the cartilage of the neck [14,29]. The maxilla and the middle and largest part of the mandible will undergo intramembranous ossification to directly form bone, whereas, in mammals, Meckel’s cartilage will either undergo endochondral ossification to contribute to the mandible in the distal and proximal regions, or it disintegrates in the middle portion of the mandible [29]. The malleus and incus also form bone through endochondral ossification. In zebrafish, cartilage of the jaw and gills constitute the viscerocranium. The first branchial arch contributes to Meckel’s cartilage, the second arch develops into ceratohyal and hyosymplectic, and the third through seventh arches give rise to posterior ceratobranchial cartilages [5,28,30,31,32,33,34,35]. The anterior dorsal neurocranium, a supportive cartilage for the brain and auditory capsule, is derived from a stream of neural crest cells that migrate anterior to the forebrain and anterior to the midbrain [36,37]. Neural crest cells migrating anterior from the first arch contribute to the ethmoid plate or zebrafish palate, which is positioned at the anterior portion of the neurocranium, and cells migrating posterior from the first arch give rise to trabecular cartilages in the posterior neurocranium [9,37,38]. Hence, neural crest cells contribute to the craniofacial skeletal elements in both mice and zebrafish.

The specification, migration, and terminal differentiation of cNCCs is tightly controlled by numerous regulators, such as transcription factors and epigenetic regulators of the genome, such as chromatin-modifying enzymes. Epigenetic modifications, including histone acetylation and methylation, nucleosome positioning, and DNA methylation, regulate the transcription of target genes. Importantly, mutations in chromatin modifiers, such as Chd7, Mll2 (Kdm6a), and Kat6a/b, result in craniofacial defects and give rise to the human syndromes, including Charge, Kabuki, and Ohdo, respectively [39,40,41,42]. Nonetheless, even individuals with the same genotype and craniofacial syndrome can differ in phenotype and penetrance. Although the epigenetic regulation of neural crest cells has begun to be explored [43,44,45], the role of chromatin modifiers in the development of cNCCs is not yet well-defined, even though craniofacial defects are a frequent birth defect (1 in 1000 births worldwide) [46,47].

An important epigenetic modification that promotes transcription is acetylation of lysine 9 residue of histone H3 (H3K9ac) [48,49]. H3K9ac is typically associated with transcriptional activation. A recent study reported that H3K9ac mediates a switch between transcriptional initiation to elongation by releasing the paused RNA polymerase II at the proximal promoter, activating gene expression [50]. In addition, H3K9ac also interacts via cross-talk with other histone modifiers to regulate developmental gene expression [51,52,53,54]. Two epigenetic modifying enzymes known to acetylate H3K9 are Kat2a and Kat2b (also known as GCN5 and PCAF, respectively), and they are both highly conserved from yeast to human. Kat2a and Kat2b share some degree of protein homology, with 73% amino acid sequence identity and conserved domains, including a bromodomain and acetyltransferase domain [55].

Kat2a and Kat2b regulate various physiological phenomenon, including embryonic development, brown adipogenesis, transcription, genome stability, cancer, immune response, and angiogenesis [56,57,58,59,60,61]. In this study, we specifically focused on their role in early developmental stages of the craniofacial skeleton, which is not well-understood. Both Kat2a and Kat2b are implicated in several signaling pathways during development, including retinoic acid (RA), Wnt, and Notch [62,63,64]. In mouse, *Kat2a* is expressed throughout the embryo except for the allantois and the heart and maintains high expression throughout development, at least until E16.5 [65]. Complete loss of *Kat2a* in mice results in embryonic lethality by embryonic day (E) 10.5 (E10.5), while *Kat2b* homozygous mutants are developmentally normal and survive into adulthood with no obvious defects [65]. Double homozygous null mutant mice for *Kat2a/Kat2b* die in utero by E7.5, earlier than the *Kat2a* single mutants, suggesting that there is some functional overlap between these two genes early in development [65]. Creation of a catalytically inactive form of Kat2a by creating two point mutations in the histone acetyl transferase domain (HAT) results in a cranial neural tube defect and lethality of *Kat2a^hat/hat^* embryos by E16.5, suggesting that the HAT activity of Kat2a is critical for embryonic development [62,66]. Previous work from our group shows that in the mouse forebrain, upon binding of RA to its receptor RARα/γ, Kat2a directly acetylates TACC1, which represses RA-mediated signaling in the absence of RA [62]. However, embryonic in vivo studies of Kat2a mutants are limited, and Kat2a has not been implicated in murine craniofacial development. A recent study in zebrafish implicates *kat2a* and *kat2b* in heart and fin development [67]. Tbx5, a transcription factor critical for heart and forelimb development, is directly acetylated by *kat2a* and *kat2b* [67]. Knockdown of *kat2a* and *kat2b* in zebrafish results in defects in heart looping and fin outgrowth, similar to that in *tbx5* mutants, thus connecting the acetylation activity of Kat2a and Kat2b to heart and limb development [67]. However, the role of *kat2a* and *kat2b* has not been explored in zebrafish craniofacial development.

In this study, we genetically disrupted *kat2a* and *kat2b* using TALEN and CRISPR/Cas9 genome editing technologies, respectively, and analyzed the previously published *Kat2a^hat/hat^* mouse model. We observed defects in the neural crest cell-derived craniofacial cartilage and bone formed from both endochondral and intramembranous ossification, including hypoplastic cartilage and bone development in both zebrafish and mice. Our results further show a reduction in posterior ceratobranchial cartilages and a reduced expression of markers for dermal bones in zebrafish, and hypoplastic and ectopic bone growth as well as cartilage defects in mice. These results imply that mutations in *kat2a* and *kat2b* affect both pathways of ossification. We also observed differential expression of neural crest cell-specific cartilage and bone differentiation factors, including *sox9a*, *col2a1*, and *runx2a*/*2b*, as well as a reduction in H3K9ac levels in zebrafish and mouse mutant embryos. Collectively, our study reveals previously unidentified roles for the histone acetyltransferases *kat2a* and *kat2b* in craniofacial development.

## 2. Materials and Methods

### 2.1. Zebrafish

Zebrafish were maintained as previously described [68]. Wildtype (WT) strains used AB, and transgenic lines include Tg(*sox10*:EGFP) [69]. Transgenic fish were used alone or crossed with various mutant backgrounds. Developmental staging followed published standards [70]. All experiments utilizing zebrafish were approved by the University of Colorado Denver IACUC protocol #147 and conform to NIH regulatory standards of care.

### 2.2. Genotyping of Zebrafish

Fin clips or single embryos were placed in Lysis Buffer (10 mM TrisHCl (pH 8.0) 50 mM KCl, 0.3% Tween 20, 0.3% NP40, 1 mM EDTA) for 10 min at 95 °C, incubated with 50 μg of Proteinase K at 55 °C for 2–3 h, and incubated again at 95 °C for 10 min. For genotyping *kat2a* TALEN mutants, the following primers were used, (Fw) 5′-AACAGGATATTGTGAAGAAAG-3′ and (Rv) 5′-ACTCCGAGTTTCTCCAGCTT-3′, to generate a 500 bp PCR product. To identify mutant embryos, PCR product was then incubated with SacI-HF (New England BioLabs, Catalog# R3156S), which digests the WT sequence but is unable to cut the mutant sequence. Heterozygotes were identified after SacI-HF digestion, as a 500 bp band for the mutant allele and two lower bands for the WT allele, similar to digestion of WT DNA. *kat2b* CRISPR mutants were genotyped using the following primers (Fw) 5′-TACTACACGTTTGTGGCCTTT-3′ and (Rv) 5′-GCGTTTCTCAGGTGGTAGTT-3′, to generate a 200 bp PCR product. For PCR reactions, REDExtract-N-Amp™ PCR 2× ReadyMix™ (Sigma) was used and subjected to the same genotyping protocol. PCR product was then treated with enzyme T7 Endonuclease I (New England BioLabs, Catalog# M0302L, Ipswich, MA USA), which cleaves double-stranded DNA at the site of mismatch. Heterozygous and homozygous CRISPR mutants were distinguished using primers that were designed with dCAPS Finder 2.0 (helix.wustl.edu/dcaps/dcaps. html). PCR amplification on diluted PCR product from the first PCR with the primer pairs (Fw) 5′-GTTATTGTAACGTGCCCCAGTTCCG-3′ and (Rv) 5′-GCGTTTCTCAGGTGGTAGTT-3′, followed by digestion with AciI (New England BioLabs, Catalog# R0551L), yields a digested lower band for mutant, an undigested upper band for WT, and a combination of both for heterozygotes.

### 2.3. Mice and Genotyping

Mice were bred and maintained according to the animal care and use protocols approved by the Institution of Animal Care and Use Committee at the University of Colorado Denver Anschutz Medical Campus protocol #528 and the University of Colorado Boulder protocol #2590. All animal dissections were performed in accordance with NIH guidelines and standards. The *Kat2a^hat^* mice (also called *Gcn5^hat^*) were maintained on a C57/Bl6J background and kept in standard light conditions. Heterozygous males and females were used for breeding and genotyped as previously described [66]. Timed matings were performed, and the morning of the vaginal plug was considered to be embryonic day 0.5. Embryos of matching somite numbers were used for experiments.

### 2.4. Zebrafish kat2a and kat2b Mutant Generation

TALEN pairs were found using (TALE-NT) 2.0 [71], which is a freely accessible web-based tool to find TALEN pairs with editable parameters and the identification of a unique restriction site in the spacer sequence. The TALEN pairs targeted exon 1 of *kat2a* to create a stop codon and new reading frame close to the start site. TALEN pairs were synthesized using the Golden Gate method [72] using the GoldyTALEN vector [73]. GoldyTALEN constructs were subsequently linearized with SacI, and the mRNA encoding each TALEN was transcribed using the mMESSAGE mMachine T3 kit (Life Technologies). mRNAs encoding TALEN pairs were injected into the cytoplasm of 1-cell stage WT AB zebrafish embryos. F0-injected embryos were outcrossed to Tg(*sox10*:EGFP) zebrafish to check for germline transmission. F1 fish that were found to carry a mutation were then grown to adulthood, fin-clipped, and sequenced. F1 mutant adults were again outcrossed with Tg(*sox10*:EGFP) zebrafish. Identified F2 mutant adults were used for crosses to generate the heterozygous and homozygous F3–F5 embryos used in experiments.

CRISPR [74] target sites to mutate *kat2b* were identified using the ZiFiT Targeter (http://zifit.partners.org/ZiFiT/); the selected target sites were then used to make gene-specific gRNAs. gRNAs were then prepared based on the protocols described in [75]. A Cas9/sgRNA complex was formed by incubating 600 ng/μL Cas9 protein (PNA Bio) with sgRNA at room temperature for 5 min before injection into the cytoplasm of 1-cell stage WT AB zebrafish embryos along with KCl at a final concentration of 200 mM.

### 2.5. In Situ Hybridization, Alcian Blue Staining, and Alizarin Red Staining

Whole-mount RNA in situ hybridization (ISH) on zebrafish embryos was performed as previously described [76,77]. DIG-conjugated antisense probes were synthesized as previously published for the following genes: *dlx2a* [78] and *crestin* [79]. The following antisense probes were used: *col2a1* (obtained from Tatjana Piotrowski, Kansas City, MO USA) and *sox9*, *runx2a*, and *runx2b* (obtained from Jamie Nichols, Aurora, CO USA). DIG-conjugated antisense probes were synthesized from full-length transcript sequences in the pCS2+ plasmid using primers for the following genes:

*kat2a* Fw—5′-GAATTCCCTACACCGAACTCT-3′, Rv—5′-ACTGACACTGGGAAGAAA CTA-3’
*kat2b* Fw—5′-CACGTCATTGGAAACTC-3’, Rv—5′-CGCCTCATTCTTCTTTAC-3′

Following ISH, single-embryo genotyping for *kat2a* and *kat2b* mutant alleles was performed. Zebrafish embryos were incubated at 65 °C for 8 h in 300 mM NaCl to remove crosslinking events. Embryos were then processed for single embryo genotyping as above. Alcian blue and Alizarin red staining was performed as previously described [80].

Mouse embryos for ISH were fixed overnight in 4% paraformaldehyde (PFA) at 4 °C. Embryos for whole-mount ISH were then washed in PBS, dehydrated in a series of Methanol:PBS, and then stored in 100% Methanol. Whole-mount ISH on mouse embryos was performed as previously described [81]. Embryos for section in situ were cryopreserved overnight at 4 °C in 30% sucrose and then embedded by flash freezing in Tissue Tek O.C.T., and 10 μm sections were cut on a Leica CM3050S cryostat. Section in situ hybridization was performed as described [82]. In all cases, DIG-labeled RNA probes were used. The following RNA probes were used: *mRunx2* (a kind gift from Dr. David Clouthier), *mBmp4* (a kind gift from Dr. Liz Robertson), and *mSox9* (Fw: 5′-TGAACGAGAGCGAGAAGAA-3′; Rv: 5′-GATGGTCAGCGTAGTCGTATT-3′). Mouse skeletons were stained with alcian blue and alizarin red as previously described [83].

### 2.6. Immunohistochemistry and Western Blotting

Zebrafish embryos and larvae were fixed in 4% PFA overnight at 4 °C. Fixed embryos were embedded in 1.5% agar with 5% sucrose and transferred to a 30% sucrose solution in scintillation vials and incubated at 4 °C overnight. The blocks were then frozen, and a cryostat microtome was used to cut the blocks into 10–15 µm sections received on Superfrost Plus slides (12-550-15, Fisher Scientific, USA). The sections were incubated with the following primary antibodies: Anti-Histone H3K9 acetylation (C5B11, Cell Signaling Technology, Danvers, MA USA) and Anti-Histone H3K9me3 (ab8898, Abcam). Slides were processed using the normal Immunofluorescence staining protocol for sections. Slides were washed in PBS several times to remove buffer, then blocked for 30 min at room temperature in 10% serum-blocking buffer and incubated overnight at 4 °C with primary antibody in blocking buffer. The next day, slides were washed several times in PBS then incubated with secondary antibodies (Alexa Fluor 568 goat anti-rabbit, Life Technologies, USA) for 1 h at room temperature. Slides were then washed several times in PBS and cover-glass mounted with Permafluor (Thermo Scientific, USA). Fluorescence was imaged with a Zeiss Axiovert 200 microscope equipped with a PerkinElmer spinning disk confocal system and Volocity software (version 6.0, PerkinElmer, USA), which were imported into Adobe Photoshop. All images for a given figure were processed in the same way.

Mouse embryos were fixed in 4% PFA for 30 min at room temperature followed by overnight cryopreservation in 30% sucrose at 4 °C. Embryos were embedded in TissueTek O.C.T. and sectioned transversely using a Leica CM3050S cryostat. Ten micrometer (10 μm) tissue sections were cut and used for staining. Sections were blocked with 5% normal goat serum/1% BSA in PBS-Tween, then incubated with the following primary antibodies: rabbit anti-Runx2 (1:1600, Cell Signaling Tech., Cat #12556S), rabbit anti-H3K9ac (1:400, Cell Signaling Tech., Cat #9649S, Danvers, MA USA), and mouse anti-AP2 (1:50, DSHB, Cat #3B5-S). The secondary antibodies used included Alexa Fluor Goat anti-rabbit 488, Alexa Fluor Goat anti-mouse 555, and Alexa Fluor Goat anti-rabbit 555. DAPI was used to counterstain nuclei at a concentration of 0.5 μg/mL in PBS.

For Western blotting, 50 embryos of each genotype were collected and deyolked at 48 h post fertilization (hpf). Embryos were then lysed in 40 μL of RIPA buffer plus a protease inhibitor cocktail for 1 h. A Bradford protein assay was performed for protein concentration. Lysates were boiled in SDS sample buffer for 5 min at 95 °C. Samples were loaded into a 10% PAGE gel (BioRad, USA). Western blots were probed with primary and secondary antibodies against H3K9 acetylation (C5B11 and 7074S; Cell Signaling Technology) and histone H3 (9715S, Cell Signaling Technology and A0545, Sigma Aldrich, USA). Imaging was done with a BioRad Chemidoc multiplex imager. Data plotting and statistical analyses were performed in GraphPad Prism software (version 6) (La Jolla, CA USA).

### 2.7. Quantitative PCR

All RT-qPCR experiments on mouse embryos were carried out as two steps with reverse transcription of RNA preceding qPCR. Tissue dissected from embryos was flash frozen in liquid nitrogen, followed by RNA extraction with the Zymo Quick RNA kit (Zymo, Cat #R1050, Irvine, CA USA). cDNA was prepared from 1 μg of RNA with the Superscript III Reverse Transcriptase kit (Thermo Fisher, Cat #18080093). qPCR was carried out using Roche Universal Probe library Master Mix and Roche Light Cycler 480. A combination of GAPDH and GusB were used as a housekeeping control. The following primers and probes were used:

*Dlx1*: Fw 5′-CCGGAGGTTCCAACAAACT-3′; Rv 5′-TCTGGAACCCATATCTTGACCT-3′, Probe #13.
*Twist1*: Fw 5′-AGCTACGCCTTCTCCGTCT-3′; Rv 5′-TCCTTCTCTGGAAACAATGACA-3′, Probe #58.
*Runx2*: Fw 5′-TCCACAAGGACAGAGTCAGATTAC-3′; Rv 5′-TGGCTCAGATAGGAGGGGTA-3′, Probe #60.
*Bmp4*: Fw 5′-GAGGAGTTTCGATCACGAAGA-3′; Rv 5’-GCTCTGCCGAGGAGATCA-3′, Probe #89.
*Sox9*: Fw 5′-TATCTTCAAGGCGCTGCAA-3′; Rv 5′-TCGGTTTTGGGAGTGGTG-3′, Probe #101.
*Col2a1*: Fw 5′-AAGAACCAGACTGCCTCAAC-3′; Rv 5′-CCTTTGGCCCTAATTTTCG-3′, Probe #56.

### 2.8. Statistical Analysis

Measurements of zebrafish viscerocranium were completed from the anterior most aspect of Meckel’s cartilage at the midline to the base of the hyosymplectic, as previously described [78]. Similar measurements of the neurocranium were made from the ethmoid plate to the base of the trabeculae. All statistical analyses and graphing for experiments were done using either R Studio or PRISM. Statistical significance was determined using Welch’s T-Test with *p* < 0.05 considered significant.

## 3. Results

### 3.1. kat2a and kat2b Are Expressed Broadly during Zebrafish Embryogenesis

**Figure 1:**
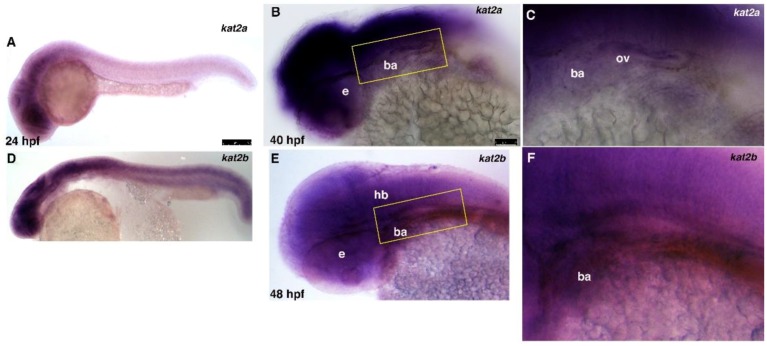
***kat2a* and *kat2b* are broadly expressed throughout the head region during zebrafish development.** (**A**–**F**) Whole-mount in situ hybridization (ISH) of *kat2a* and *kat2b* expression during zebrafish development. Wildtype zebrafish embryos express both *kat2a* and *kat2b* in the head region, including the brain, the branchial arches, and the eye. (**A**,**D**) Lateral views at 24 h post fertilization (hpf) of expression of *kat2a* and *kat2b*. (**B**,**C**) Lateral views of wildtype embryos at 40 hpf shows *kat2a* expression in the eye, the branchial arches, and the otic vesicle. (**E**,**F**) Dorsal view at 48 hpf shows *kat2b* expression in the eye, the branchial arches, and the hindbrain. Lateral views, anterior is to the left. Yellow rectangles in B and E represent the regions shown in C and F, respectively. e, eye; ba, branchial arches; hb, hindbrain; ov, otic vesicle. Scale bars represent 250 μm (**A**,**D**) and 100 μm (**B**,**E**).

To determine the expression of *kat2a* and *kat2b* within the cranial neural crest region during zebrafish development, we performed in situ hybridization between 24 and 48 h post fertilization (hpf; Figure 1). Both *kat2a* and *kat2b* are broadly expressed throughout the anterior head region, including the central nervous system (CNS), the eye, and branchial arches at 24 hpf (Figure 1A,D). *kat2a* and *kat2b* are expressed strongly in the brain region, with *kat2b* expression extending posteriorly in the spinal cord (Figure 1A,D). By 40–48 hpf, expression of both genes remains strongly expressed in the head region but is reduced throughout the rest of the embryo (Figure 1B,C,E,F). A previous analysis in mouse embryos has shown that both *Kat2a* and *Kat2b* are similarly ubiquitously expressed [65], and importantly they are expressed in the branchial arch mesenchyme. A cross-sectional analysis confirms that zebrafish *kat2a* is broadly expressed in the brain, the lens of the eye, and the cranial mesenchyme where neural crest cells are condensing to form cartilage and bone (not shown). Thus, *kat2a* and *kat2b* are expressed in the right place and time in both zebrafish and mouse embryos to be involved in craniofacial development.

### 3.2. Loss of kat2a and kat2b in Zebrafish, and Kat2a^hat/hat^ in Mice, Results in Craniofacial Cartilage and Bone Defects

To determine the function of *kat2a* and *kat2b* in cNCC development, we created mutants in zebrafish and analyzed the existing *Kat2a*^hat/hat^ mouse mutant. For the zebrafish mutations, we used both TALEN- and CRISPR/Cas9-based mutagenesis. *kat2a* zebrafish mutants were created with TALEN gene-targeted mutagenesis to generate two mutant *kat2a* alleles (Supplemental Figure S1A–C). The TALEN targeted exon 1 to create a stop codon and a new reading frame close to the start site (Schematic in Supplemental Figure S1A). We then outcrossed the injected F0 adult zebrafish, sequenced the locus in the resulting embryos, and identified two independent *kat2a* alleles that are predicted to create severe loss-of-function mutations with short open reading frames (ORFs; 303–353 base pairs). These lines were maintained for further analysis and designated *kat2a*^CO1007^ and *kat2a*^CO1010^, with *kat2a*^CO1007^ used for the remainder of this study (referred to as *kat2a*^−/−^ mutants from this point forward). *kat2b* zebrafish mutants were generated using CRISPR/Cas9 (Supplemental Figure S1D–F). Guide RNAs were designed to target a Protospacer adjacent motif (PAM) site in exon 6 and we identified three alleles by sequencing: *kat2b*^CO1008^, *kat2b*^CO1011^, and *kat2b*^CO1012^ with an ORF for the mutations between 858 and 990 base pairs. *kat2b*^CO1008^ was used for the remainder of this study (referred to as *kat2b*^−/−^ mutants from this point forward). To determine whether the expression of *kat2a* and *kat2b* was reduced in the respective zebrafish mutants, in situ hybridization was performed. Comparing wildtype *kat2a* and *kat2b* expression in *kat2a*^−/−^ and *kat2b*^−/−^ embryos, respectively, there is a clear reduction in expression in the mutant embryos (Supplemental Figure S2). Hence, although the *kat2b* alleles have mutations in a later exon, the resulting mutations are predicted to be loss-of-function alleles.

**Figure 2:**
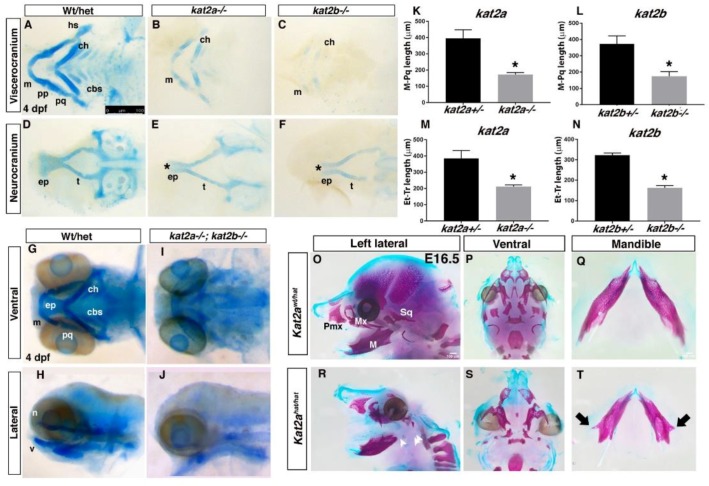
**Loss of *kat2a* and *kat2b* causes defects in craniofacial development. Alcian blue staining** to detect cartilage formation in 4 dpf (days post fertilization) wildtype/heterozygote zebrafish embryos (**A**,**D**,**G**,**H**), *kat2a^−/−^* (**B**,**E**), *kat2b*^−/−^ (**C**,**F**), and *kat2a*^−/−^;*kat2b*^−/−^ double mutant zebrafish embryos (**I**,**J**). (**A**–**F**) Cartilage-stained embryos were dissected to separate the viscerocranium and the neurocranium. As compared to the control (**A**,**D**), the flat-mounted 4 dpf *kat2a* (**B**,**E**) and *kat2b* (**C**,**F**) mutant larvae have a reduced palatoquadrate, including the pterygoid process of the palatoquadrate and the hyosymplectic of the viscerocranium, shortening of the Meckel’s cartilage, widening of the angle between ceratohyals in the viscerocranium, a smaller hypoplastic ethmoid plate, and deformed trabeculae of the neurocranium, with hypoplastic ceratobranchial cartilages that are also reduced in number. In the *kat2a and kat2b* mutant embryos, there is a gap in the anterior edge of the ethmoid plate that forms a “cleft” as shown (*) in (**E**,**F**). Cartilage defects were observed in n = 17/62 for *kat2a* and n = 16/66 for *kat2b*, with the total number of mutant embryos being n = 20/72 for *kat2a* and n = 17/70 for *kat2b* in each clutch. (**G**–**J**) Ventral and lateral views of whole-mount, alcian-blue-stained cartilage of control (**G**,**H**) and *kat2a^−/−^*;*kat2b*^−/−^ double mutants (**I**,**J**). (**K**–**N**) Quantitation of length in micrometers (µm) from Meckel’s cartilage to palatoquadrate (M–Pq), and from ethmoid plate to trabeculae (Et–Tr), in *kat2a* and *kat2b* mutants shows a significant reduction in length. * denotes statistical significance (*p* < 0.05) using Welch’s T-Test. Error bars show the standard error of the mean. (**O**–**T**) Whole-mount E16.5 *Kat2a^Wt/hat^* (**O**–**Q**) and *Kat2a^hat/hat^* (**R**–**T**) embryo skeletons stained with alcian blue for cartilage and alizarin red for bone. (**O**,**R**) Left lateral view of skull. (**P**,**S**) Ventral view of skull with the mandible dissected off. (**Q**,**T**) Dorsal view of the dissected mandible. The arrows in T point to ectopic bone growth on the mandible (ectopic bone growth observed in n = 3). The arrow heads in R point to the missing first arch elements. cb, ceratobranchials; ch, ceratohyal; e, ethmoid plate; hs, hyosymplectic; Mx, Maxilla, M, Mandible; m, Meckel’s cartilage; n, neurocranium; pp, pterygoid process; pq, palatoquadrate; Pmx, Pre-maxilla; Sq, Squamosal; t, trabeculae; v, viscerocranium. The scale bars represent 100 μm.

To test the hypothesis that *kat2a* and *kat2b* play a role in cartilage development, we assessed cartilage formation in the *kat2a*^−/−^ and *kat2b*^−/−^ zebrafish embryos by performing Alcian blue staining at 4 days post fertilization (dpf). Both zebrafish mutants display severe craniofacial defects (Figure 2A–F,K–N). *kat2a*^−/−^ embryos display overall hypoplastic or loss of cartilage elements, including a reduced palatoquadrate, a reduced pterygoid process of the palatoquadrate and hyosymplectic of the viscerocranium, shortening of the Meckel’s cartilage, and widening of the angle of the ceratohyal of the viscerocranium. *kat2a*^−/−^ embryos also have a smaller hypoplastic ethmoid plate, a deformed trabeculae of the neurocranium, and ceratobranchials that are reduced in number with the anterior elements hypoplastic if present. *kat2b*^−/−^ embryos have a similar but more severe hypoplasia, including a complete lack of staining of the posterior ceratobranchial cartilages, and a more significant gap in the anterior edge of the ethmoid plate that forms a “cleft” (Figure 2E,F). Hence, several cartilage elements of the craniofacial skeleton, including the neurocranium, ceratohyal, and ceratobranchials, which are formed via endochondral ossification, are affected in *kat2a*^−/−^ and *kat2b*^−/−^ embryos. Quantification of the length from Meckel’s cartilage to the palatoquadrate and from the ethmoid plate to the tribecule indicates a significant reduction by more than 50% in both mutants when compared to the wildtype (Figure 2K–N). Interestingly, *kat2a* heterozygous embryos also display a mild phenotype, which suggests that the created mutation is a dominant mutation (~19% of heterozygous embryos). Both *kat2a*^−/−^ and *kat2b*^−/−^ were viable at 4 dpf, presenting at ~25% of the total number of embryos in each clutch. However, for both single zebrafish mutants, the embryos did not survive to 6–7 dpf, and, therefore, we were unable to assay for bone formation.

To determine if *kat2a* and *kat2b* function together or redundantly in craniofacial cartilage development, we created double *kat2a*^−/−^;*kat2b*^−/−^ mutant zebrafish embryos and examined the craniofacial phenotype at 4 dpf (Figure 2G–J). Alcian staining of double *kat2a*^−/−^;*kat2b*^−/−^ mutant embryos showed a more severe craniofacial phenotype compared to the individual *kat2a*^−/−^ and *kat2b^−/−^* mutants. In fact, because of the reduction in cartilage formation, it was not possible to dissect the viscerocranium from the neurocranium. The whole-mount stains of double *kat2a^−/−^*;*kat2b*^−/−^ mutants suggest that the cartilage elements start to differentiate but then differentiation stalls, since only a small portion of the neurocranium and the early developing viscerocranium has begun to form (Figure 2G–J). It has been described previously that zebrafish cartilage develops in a specific order, and the pattern we observe is consistent with that analysis, as shown by morphology [28] as well as gene expression [84]. These data indicate that, in addition to sharing redundant roles, *kat2a* and *kat2b* also contribute individually to craniofacial development.

To determine whether mouse Kat2a also functions in craniofacial development, we analyzed skeletal development in the *Kat2a^hat/hat^* mouse homozygous mutants. This also allowed us to extend our study to craniofacial bone formation. Previous work from our lab and others has identified that the HAT activity of Kat2a is critical for development; however, a role for Kat2a in craniofacial development has not been previously identified. Interestingly, previous *in vitro* studies using mesenchymal stem cells that were differentiated into osteoblasts and treated with shRNA to knock down Kat2a showed that disruption of Kat2a resulted in a loss of *Runx2* expression [85], raising the question of whether Kat2a regulates bone growth *in vivo*. Therefore, to test the hypothesis that Kat2a is required for skeletal development, we evaluated bone and cartilage formation in E16.5 wildtype and *Kat2a^hat/hat^* mouse mutants using Alizarin Red and Alcian blue staining. This revealed several craniofacial phenotypes in the *Kat2a^hat/hat^* mouse mutants. Of these, the most severe defects were observed in derivatives of the mandibular prominence of the first branchial arch, affecting both endochondral and intramembranous ossification of the craniofacial skeleton. Intramembranous bone defects included ectopic bone growth on the mandible or dentary bone (Figure 2T, black arrows), as well as a hypoplastic squamosal (Figure 2R). Endochondral ossification defects included loss of mandibular condyles, hypoplastic malleus and incus, as well as missing tympanic rings of the ear (Figure 2R, arrow heads). In the *Kat2a^hat/hat^* mutants, we did not observe a cleft lip nor a palate. We also did not observe any skeletal defects in the axial skeleton (data not shown). Thus, the *kat2a/kat2b* acetyltransferase genes are required in both zebrafish and mouse for proper craniofacial development.

### 3.3. Cartilage and Bone Markers are Differentially Expressed in kat2a and kat2b Zebrafish and Kat2a^Hat/Hat^ Mouse Embryos

The cartilage and bone defects in *Kat2a^hat/hat^* mutant mouse embryos led us to first examine whether these phenotypes were due to a defect in cNCC specification or branchial arch patterning. To do so, we examined the expression of AP2, *Twist1*, and *Dlx1* in E9.5 and E10.5 mouse embryos. *Ap2* is expressed in the ectoderm and migratory cNCCs and acts to specify cNCC identity, while *Twist1* and *Dlx1* are expressed in the branchial endoderm and regulate branchial arch patterning [86]. At both E9.5 and E10.5, AP2 positive migratory cNCCs can be seen in the branchial arches of *Kat2a^hat/hat^* and control mouse embryos, suggesting that cNCC specification is not affected by loss of Kat2a HAT activity in mice (Supplemental Figure S3A–H). Similarly, the expression of *Dlx1* was not significantly changed in E9.5 and E10.5 *Kat2^hat/hat^* mutants compared to control mouse embryos (Supplemental Figure S3J,K). We did observe a significant change in the expression of *Twist1* in E9.5 *Kat2a^hat/hat^* mutant mouse embryos; however, this change was not observed in E10.5 mutants compared to the control (Supplemental Figure S3J,K). Likewise, in *kat2a^−/−^* and *kat2b^−/−^* zebrafish embryos, expression of *dlx2* and *crestin* was not significantly reduced at early stages of neural crest cells development (Supplemental Figure S4). Together, these results suggest that *Kat2a/2b* is required after cNCC specification and migration.

**Figure 3:**
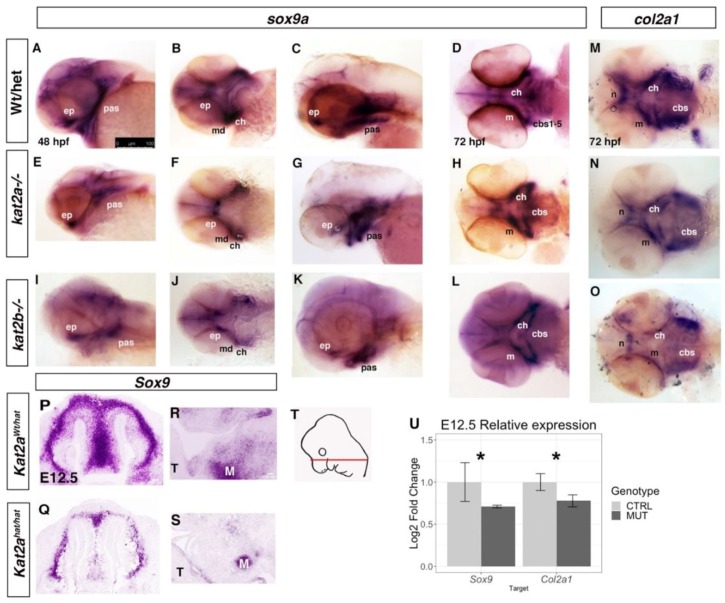
**Cartilage progenitor expression of *sox9* and *col2a1* is reduced in *kat2a^−/−^* and *kat2b^−/−^* zebrafish embryos and E12.5 *Kat2a^hat/hat^* mice.** Whole-mount in situ hybridization for zebrafish *sox9a* (**A**–**L**) and *col2a1* (**M**–**O**) in wildtype or heterozygous embryos (**A**–**D**,**M**), *kat2a^−/−^* (**E**–**H**,**N**), and *kat2b^−/−^* (**I**–**L**,**O**) zebrafish embryos at 48 hpf (**A**,**B**,**E**,**F**,**I**,**J**) and 72 hpf (**C**,**D**,**G**,**H**,**K**–**O**). Lateral views (**A**,**C**,**E**,**G**,**I**,**K**), and ventral views (**B**,**D**,**F**,**H**,**J**,**L**–**O**), with the anterior to the left in all cases. At both stages, *sox9a* and *col2a1* expression is reduced in the forming cartilage elements. At 48 hpf, there is an overall reduction, especially in the anterior expression domain of *sox9a* (**E**, **F** and **I**, **J** as compared to control (**A**,**B**)). At 72 hpf, the number of cartilage elements is reduced, especially in the ceratobranchial cartilages in both *kat2a* (**G**,**H**,**N**) and *kat2b* (**K**,**L**,**O**) as detected with both *sox9a* and *col2al*. For *sox9*, n = 8/33 for *kat2a*, n = 7/30 for *kat2b*. For *col2a1*, n = 7/29 for *kat2a*, n = 7/31 for *kat2b*. (**P**–**S**) Sectional in situ hybridization with a *Sox9* antisense probe on mouse E12.5 *Kat2a^Wt/hat^* (**P**–**R**) and *Kat2a^hat/hat^* (**Q**–**S**) cryosections through the frontonasal prominence (**P**,**Q**) and the mandibular prominence (**R**,**S**) as schematized in the diagram of a E12.5 embryo adapted from the eHistology Atlas (T, the red bar indicates the plane of transverse sections). M = Meckel’s cartilage, T = Tongue. (**U**) RT-qPCR quantification of *Sox9* and *Col2a1* expression with RNA extracted from the facial prominences of control (*Kat2a^Wt/hat^*; CTRL on graph) and mutant (*Kat2a^hat/hat^*; MUT on graph) E12.5 mouse embryos. Expression was normalized to GusB, n = 4. The Y-axis shows the Log2 fold change of the relative gene expression, following the ddCT method; * denotes statistical significance (*p* < 0.05) using Welch’s *t*-Test. The error bars show the standard error of the mean. cbs1-5, ceratobranchials 1-5; ch, ceratohyal; ep, ethmoid plate; m, Meckel’s cartilage; n, neurocranium; pas, pharyngeal arches. The scale bars represent 100 μm.

**Figure 4:**
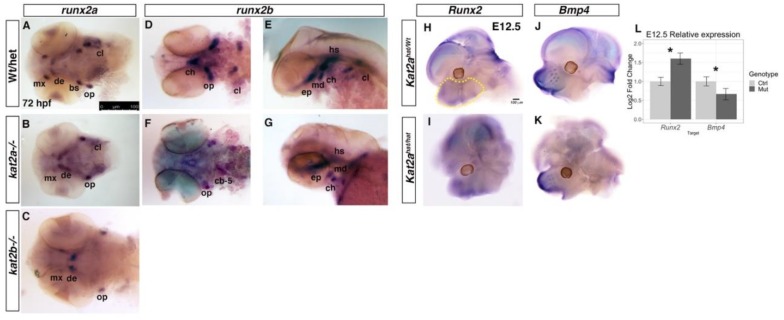
**The bone progenitor markers *runx2a* and *runx2b* are differentially expressed in *kat2a^−/−^* and *kat2b^−/−^* zebrafish embryos and *Kat2a^hat/hat^* mouse embryos.** In situ hybridization for *runx2a* (**A**–**C**) and *runx2b* (**D**–**G**) in WT or heterozygous (**A**,**D**,**E**), *kat2a^−/−^* (**B**,**F**,**G**), and (**C**) *kat2b^−/−^* 72 hpf zebrafish embryos. (**A**–**D**,**F**) ventral views, (**E**,**G**) lateral views, with the anterior to the left in all. *kat2a^−/−^* (**B**) and *kat2b^−/−^* (**C**) show reduced expression of *runx2a*, especially in the anterior maxilla, whereas expression in the dentary bone remains strong as compared to controls (**A**). *runx2b* expression in forming bone elements is reduced overall in *kat2a* mutants (**F**,**G**) compared to the control (**D**,**E**). For *runx2a*, n = 9/34 for *kat2a*, n = 8/31 for *kat2b*. For *runx2b*, n = 8/30 for *kat2a*, n = 8/29 for *kat2b*. (**H**–**L**) Whole-mount in situ hybridization with *Runx2* and *Bmp4* antisense probes on mouse E12.5 *Kat2a^Wt/hat^* (**H**,**J**) and *Kat2a^hat/hat^* (**I**,**K**) embryos. (**L**) RT-qPCR quantification of *Runx2* and *Bmp4* expression with RNA extracted from E12.5 facial prominences of control (*Kat2a^Wt/hat^*; Ctrl on graph) and mutant (*Kat2a^hat/hat^*; Mut on graph) embryos. The yellow outline in (**H**) marks the area dissected for RNA extraction. Gene expression was normalized to GusB, n = 4. The Y-axis shows the Log2 fold change of the relative gene expression, following the ddCT method; * denotes statistical significance (*p* < 0.05) using Welch’s *t*-Test. The error bars show the standard error of the mean. Neural tube closure defects are evident in *Kat2a^hat/hat^* mutants. bs, branchiostegal ray; cb-5, ceratobranchial-5; ch, ceratohyal; cl, cleithrum; de, dentary; ep, ethmoid plate; hs, hyosymplectic; md, mandible; mx, maxilla; op, opercle. The scale bars represent 100 μm.

To determine whether Kat2a/Kat2b are involved more directly in regulating craniofacial bone and cartilage development, we investigated the temporal pattern of gene expression for cartilage and bone in the zebrafish and mouse mutants. To assess pre-chondrocyte differentiation, we used in situ hybridization to examine the expression of *sox9* and *col2a1* between 48 and 72 hpf in zebrafish embryos. In zebrafish, the overall level of *sox9a* was reduced in both the *kat2a^−/−^* and *kat2b^−/−^* mutants (Figure 3A–L). Moreover, the domains of *sox9a* expression were also smaller in accordance with the overall smaller head size as compared to wildtype or heterozygous siblings at the same time point (Figure 3A–L). *sox9a* expression at 48 hpf marks the early forming cartilage of the Meckel’s cartilage and the palatoquadrate, whereas in the *kat2a* and *kat2b* mutant embryos, the anterior Meckel’s cartilage expression appears to be diffused, and the expression in the posterior elements is significantly reduced compared to the corresponding wildtypes (Figure 3E,F,I,J compared to WT in Figure 3A,B). In 72 hpf wildtype zebrafish, when the cartilage elements are beginning to form, *sox9a* and *col2a1* are expressed within the forming Meckel’s cartilage, the ceratohyal, and the ceratobranchial cartilages 1–5 (Figure 3C,D,M). However, in the *kat2a* and *kat2b* mutant zebrafish embryos, we observed a delay in formation of the cartilages and *sox9a* and *col2a1* expression, with *sox9a* (Figure 3G,H,K,L) and *col2a1* (Figure 3N,O) expression present in the ceratohyal as well as the first ceratobranchial. We were unable to assay for hypertrophic chondrocyte markers that are expressed at later developmental stages because the zebrafish mutants do not survive up to those stages. To determine the effects on bone formation in the absence of *kat2a* and *kat2b*, we performed in situ hybridization with the markers of bone precursors *runx2a* and *runx2b* at 72 hpf. In *kat2a* and *kat2b* mutant embryos, *runx2a* and *runx2b* expression is reduced (Figure 4). While each element is present in the *kat2a* and *kat2b* mutants, the expression is significantly decreased in the maxilla, ceratohyal, ceratobranchials, and cleithrum (Figure 4B,C,F,G) as compared to wildtype embryos at the same stage (Figure 4A,D,E). There is one exception, where we observe an increase in *runx2a* expression in the forming dentary bone (Figure 4B,C as compared to Figure 4A). The decrease in the expression of bone precursors in the mutants in the maxilla, branchiostegal ray, and opercle, which are formed via intramembranous ossification, indicate that *kat2a* and *kat2b* mutations affect this pathway, in addition to the endochondral ossification described previously (Figure 2A–F). Combined, these results suggest that *kat2a* and *kat2b* is required for cartilage and bone differentiation in zebrafish.

In E12.5 mouse embryos following condensation of the cNCC-derived mesenchyme, *Sox9* RNA expression within the facial prominences was visibly decreased in the frontonasal prominence of *Kat2a^hat/hat^* embryos compared to controls (Figure 3P,Q), although *Sox9* expression was detected in Meckel’s cartilage (Figure 3R,S). Quantification of the expression of *Sox9* and *Col2a1* in *Kat2a^hat/hat^* and control mouse embryos by RT-qPCR using RNA extracted from E12.5 facial prominences showed that both *Sox9* and *Col2a1* were significantly decreased in expression in mutant mouse embryos (Figure 3U). We also assayed the expression of the osteoblast progenitors *Bmp4* and *Runx2* by whole-mount in situ hybridization of E12.5 mouse embryos. *Kat2a^hat/hat^* mutant mouse embryos showed no obvious loss of expression of *Bmp4* or *Runx2* compared to controls (Figure 4H–K). We quantified the expression of these markers using RNA extracted from the facial prominences of E12.5 *Kat2a^hat/hat^* and control mouse embryos followed with RT-qPCR quantification. This showed a significant increase in the expression of *Runx2*, but also a significant reduction in *Bmp4* in *Kat2a^hat/hat^* mouse mutants (Figure 4L). This may suggest that a subpopulation of osteoblasts is increased in *Kat2^hat/hat^* mice, perhaps related to ectopic bone formed through intramembranous ossification (Figure 2T) and similar to the increased dentary bone expression of *runx2a/2b* in zebrafish. Taken together, our data support a role for Kat2a in both mouse and zebrafish chondrocyte and osteoblast development.

### 3.4. H3K9ac Is Reduced in Kat2a and Kat2b Mutants

**Figure 5:**
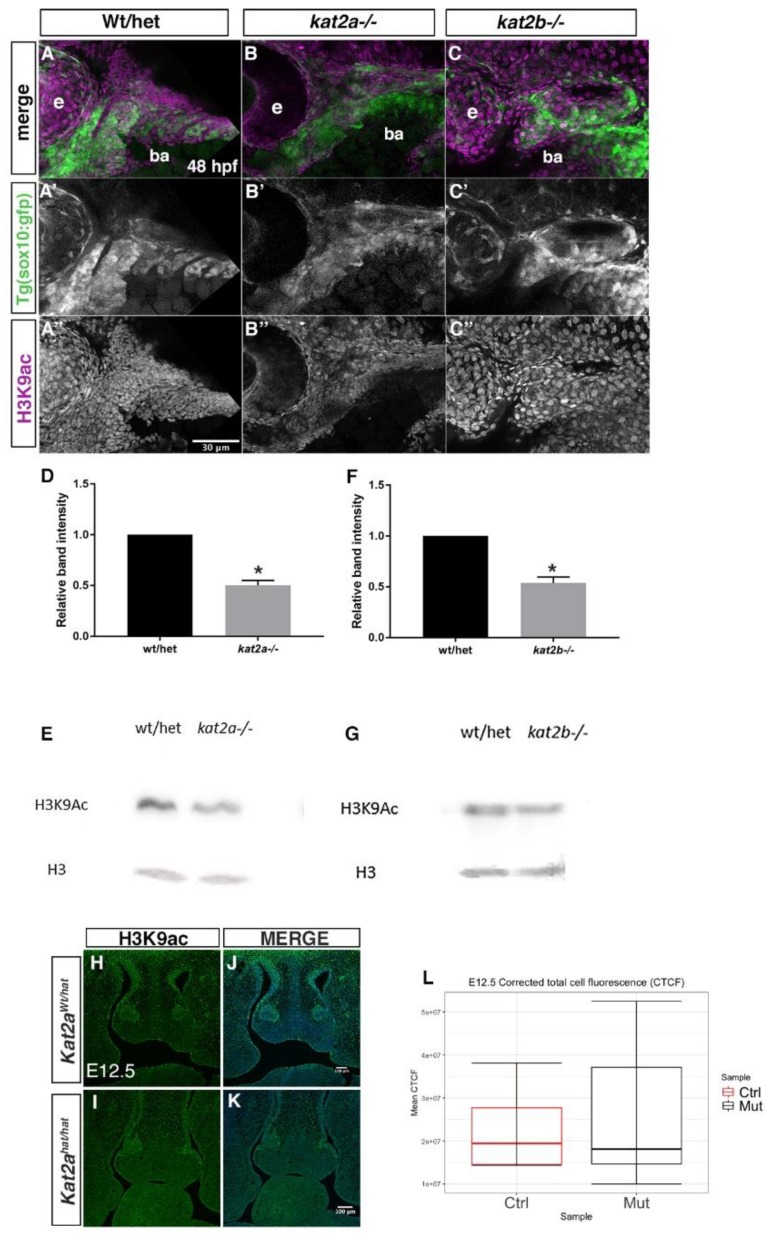
Histone H3 lysine 9 acetylation (H3K9Ac), an active transcription mark, is reduced in *kat2a^−/−^* and *kat2b^−/−^* zebrafish. (**A**–**C**″) Maximum projection confocal micrographs of 48 hpf zebrafish embryos labeled to detect H3K9Ac immunofluorescence (**A**″–**C**″, magenta in **A**–**C**) and neural crest cells expressing transgenic *sox10*:GFP (**A**′–**C**′, green in **A**–**C**). Lateral views with the anterior to the left; e, eye; ba, branchial arches. *kat2a^−/−^* and *kat2b^−/−^* zebrafish mutants show an overall disorganization of neural crest cells in the branchial arches as well as a reduction of H3K9Ac. (**B**,**C**) as compared to control (**A**). Western blots and quantification for H3K9Ac in *kat2a^−/−^* (**D**,**E**) and *kat2b^−/−^* (**F**,**G**) using 48 hpf zebrafish whole embryos relative to total histone H3 expression; * denotes statistical significance (*p* < 0.05) using Welch’s *t*-Test. The error bars show the standard error of the mean. (**H**–**K**) Immunofluorescence with the H3K9ac antibody and a DAPI counterstain on mouse E12.5 *Kat2a^Wthat^* control (**H**,**J**) and *Kat2a^hat/hat^* mouse mutant (**I**,**K**) cryosections through the frontonasal prominence, with merged images of H3K9ac and DAPI (**J**,**K**). (**L**) Quantification of corrected total fluorescence cell intensity (CTCF). Total fluorescence was corrected for background and area; Measurements were made using ImageJ, n = 2. *Kat2a^Wt/hat^* is Ctrl on graphs, *Kat2a^hat/hat^* is MUT on graphs. No significance was found for the mouse data. The scale bars represent 30 μm for zebrafish and 100 μm for mice.

We next tested the hypothesis that Kat2a/Kat2b regulates the expression of bone and cartilage specifiers through H3K9 acetylation, which is associated with the activation of transcription. To begin to address this, we analyzed the H3K9ac expression in both zebrafish and mouse during craniofacial development. In zebrafish embryos at 48 hpf, when neural crest cells have populated the branchial arches and begun to differentiate, we analyzed H3K9ac both by a Western blot analysis and immunofluorescence staining (Figure 5). In *kat2a^−/−^* and *kat2b^−/−^* zebrafish at 48 hpf, neural crest cells expressing *sox10*:GFP, while present in the branchial arches, are reduced in number and highly disorganized such that each arch is not defined (Figure 5A′–C′). To assay for H3K9 acetylation (AC), we stained these embryos with an antibody that recognizes this specific H3K9 acetyl mark in vivo. H3K9Ac levels were reduced in branchial arch regions where migrating cranial neural crest cells populate and differentiate into craniofacial derivatives (Figure 5A–C″). This is in particular observed in *kat2a* mutant zebrafish embryos (Figure 5B). In addition, this reduction is observed in the more anterior migrating cells that migrate to a dorsal position of the yolk ball, likely forming the cardiac neural crest populations (Supplemental Figure S5). To confirm and quantify the levels of H3K9Ac in *kat2a^−/−^* and *kat2b^−/−^* whole zebrafish embryos, we used a Western blot analysis. Similarly to what we observed in whole-mount immunofluorescence staining, H3K9Ac was significantly reduced at 48 hpf when compared to the wildtype (Figure 5D–G; quantified as relative intensity compared to total H3 levels). These data suggest that *kat2a* and *kat2b* function as histone acetyltransferases during craniofacial development in zebrafish.

We also tested H3K9ac expression in mouse using immunofluorescence on cryosections through the craniofacial region in E12.5 mouse embryos, when bone and cartilage specification is taking place. At E12.5, we did not observe a significant change in the overall staining in *Kat2a^hat/hat^* mutant mouse embryos as compared to *Kat2a^Wt/hat^* control embryos in the frontonasal prominence (Figure 5H–K). Quantification of total cell fluorescence, corrected for background and area (Corrected Total Cell Fluorescence, CTCF) also did not show a significant difference in the CTCF means of E12.5 *Kat2a^hat/hat^* mouse mutants compared to controls (Figure 5L). Our data in zebrafish suggest that the craniofacial defects could be due to broad epigenetic regulation; however, in mouse global H3K9ac, levels were not significantly changed, suggesting that Kat2a may act on more specific epigenetic targets that are not detectable when viewed in whole cells and tissues.

## 4. Discussion

The epigenetic modifiers Kat2a and Kat2b are highly conserved and participate in a variety of developmental pathways. Here, we describe a previously unknown role for Kat2a and Kat2b in craniofacial development. These two acetyltransferase enzymes share a high degree of protein homology, both at the level of amino acid sequence and protein structure, and they are both broadly expressed during development. Despite this, their functions in vivo are not necessarily redundant. Murine embryos that are homozygous null for *Kat2a* die early in embryonic development; however, *Kat2b* null mice survive into adulthood. Although *Kat2b* mice have not been reported to have a craniofacial defect, here we show that both Kat2a and Kat2b are required for zebrafish craniofacial development, and the phenotype increases in severity in double Kat2a/2b mutants. Craniofacial skeletal elements belonging to both endochondral and intramembranous ossification pathways are affected in *kat2a* and *kat2b* mutants. The differences in Kat2b loss-of-function phenotypes between zebrafish and mouse could be a result of divergent evolution. Here, we also show that the HAT activity of at least Kat2a is required for murine craniofacial development, using an allele in which Kat2a protein is still made and can be incorporated into multi-protein complexes but lacks enzymatic activity. Finally, our results suggest that Kat2a acetyltransferase activity is evolutionarily conserved in craniofacial development. Simultaneously, we acknowledge that owing to their high degree of conservation across species, Kat2a and Kat2b are likely to regulate other skeletal structures, in addition to the craniofacial development reported here. Their epigenetic acetyltransferase roles may also be exerted again later in life with the onset of age-related skeletal diseases, which is yet to be determined.

The craniofacial defects that result from loss of function of Kat2a/b acetyltransferases affect the development of craniofacial elements that are derived from the first and second branchial arches in both mouse and zebrafish. These include hypoplastic Meckel’s cartilage and loss of cartilaginous elements. Our analysis of *Kat2a^hat/hat^* mutant mouse embryos at early stages of cNCC specification, migration, and arch patterning did not detect an obvious change, whereas we did observe a reduction in the expression of the chondrocyte markers *Sox9* and *Col2a1* in both zebrafish and mouse mutants. Thus, our data point to a requirement for Kat2a/Kat2b during craniofacial chondrocyte development subsequent to NCC migration and arrival in the branchial arches. Our analysis of the bone markers *Runx2* and *Bmp4* also showed a significant expression change in mouse E12.5 *Kat2a^hat/hat^* mutant embryos, suggesting that Kat2a could also have a role in both mechanisms of bone formation, requiring further studies. Previous *in vitro* studies using mesenchymal stem cells that were induced to differentiate into osteoblasts that were treated with shRNA to knockdown *Kat2a* function suggested that *Kat2a* is required for pre-osteoblast differentiation, supported by a reduction in *Runx2* expression [85]. In this study, we show that *Runx2* expression was not decreased in mouse mutants but instead increased in the craniofacial region, suggesting that pre-osteoblast differentiation is not lost. Furthermore, we did not observe abnormalities in axial skeleton development in the mouse *Kat2a^hat/hat^* mutants. One possible explanation is that *Kat2b* may be able to compensate for a loss of *Kat2a* in the axial skeleton. It is also possible that *Kat2a* has different targets in the craniofacial region compared to long bone formation in the limbs and that these targets in the craniofacial region are required for proper skeletal patterning. Nevertheless, future investigations should identify the direct targets of *Kat2a* during intramembranous and endochondral ossification.

Our findings in zebrafish and mouse are largely similar, but the differential requirement for Kat2b in zebrafish craniofacial development may be explained by a loss of conservation in the Kat2b function. As the genome of zebrafish has undergone a genome duplication event, the redundant functions of Kat2a and Kat2b could be more critical for zebrafish embryonic development. Interestingly, in zebrafish, *kat2b* has a more severe cartilage phenotype as compared to *kat2a* mutants, even though *kat2a* mutants have a more severe reduction in H3K9 acetylation. This suggests that, although *kat2a* has more enzymatic functional activity, *kat2b* may function independently of this activity for proper craniofacial development. In mouse, *Kat2b* expression is low throughout development while *Kat2a* expression is high through most of the embryonic development and then significantly drops at E16.5, with low levels detected in the adult. It has also been suggested that *Kat2b* expression is higher in the adult than in embryos, supporting the hypothesis that Kat2b may have critical functions in post-natal mammals [65]. Taken together, our results suggest that Kat2b is more critical for zebrafish development than for mouse development.

In addition to H3K9 acetylation, both Kat2a and Kat2b acetylate other non-histone substrates in various aspects of development and disease, including brain development and cancer [62,87]. However, Kat2b targets have been linked to cell differentiation, cell proliferation, and apoptosis, which may explain the severity in the cartilage phenotype in *kat2b^−/−^* versus *kat2a^−/−^* (Figure 2A–F), even though the H3K9 acetylation levels of *kat2b^−/−^* were higher (53.6%) than those of *kat2a^−/−^* embryos (50.3%) (Figure 5D,F). One promising substrate of Kat2b in this process is Gli1, which has been identified as a Kat2b binding and acetylation target in hepatocellular carcinoma cells [88]. Gli1 is a transcription factor that is required for differentiation of neural crest cells [89,90], and the acetylation of Gli1 by Kat2b may be one of the potential contributors to the *kat2b^−/−^* cartilage phenotype. Another substrate of Kat2b, Akt1, has been found to be correlated to the regulation of neural crest cells specification and migration [91] and embryonic skeletal development [92], and mutated in patients with craniofacial defects, including mandible and frontal bone deformities [93]. In addition, acetylation of Akt1 by Kat2b has been shown to regulate cell proliferation and survival *in vitro* [61,94,95]. Thus, it is clear that Kat2b regulates other factors directly, and not via the modification of H3K9. It remains to be tested whether the acetylation by Kat2b regulates the survival of chondrocytes and osteoblasts in a similar way to regulate craniofacial development.

Nonetheless, *Kat2a* and *Kat2b* are highly conserved epigenetic modifiers that preferentially acetylate H3K9. This epigenetic mark is correlated with active transcription, a critical step during cell differentiation. In the *kat2a/b* zebrafish mutants, we observed a significant reduction in the expression of H3K9ac. Although we did not observe a global change in the levels of H3K9ac in E12.5 *Kat2a^hat/hat^* mutant mouse embryos, we cannot rule out the possibility that there are changes at a single cell resolution or at specific target loci. While it should be noted that both Kat2a and Kat2b can acetylate non-histone proteins, the loss of H3K9ac, as well as the loss of expression of bone and cartilage genes, suggests that Kat2a/Kat2b are required for active transcription of key craniofacial skeletal genes. Our results support the hypothesis that Kat2a/Kat2b directly target the loci of bone- and cartilage-specific genes to activate their transcription in the craniofacial-derived mesenchyme.

## Supplemental Materials

The following are available online. Figure S1: TALEN and CRISPR mutations created in *kat2a* and *kat2b*, respectively; Figure S2: Expression of *kat2a* and *kat2b* is reduced in zebrafish mutants; genotyping strategy for *kat2a* and *kat2b* mutants, heterozygotes, and wildtype; Figure S3: cNCC specification and early branchial patterning are unchanged in E9.5 and E10.5 *Kat2a^hat/hat^* mouse mutants; Figure S4: *crestin* and *dlx2* expression are slightly reduced in *kat2a^−/−^* and *kat2b^−/−^*; Figure S5: H3K9Ac is reduced in *kat2a^−/−^* zebrafish mutants.
