# Supplementary material for: Kat2a and Kat2b Acetyltransferase Activity Regulates Craniofacial Cartilage and Bone Differentiation in Zebrafish and Mice
Source: J Dev Biol. 2018 Nov 12;6(4):27. doi: 10.3390/jdb6040027 (PMC6315545; doi:10.3390/jdb6040027)
Supplement: Supplementary file 1 [file jdb-06-00027-s001.pdf]

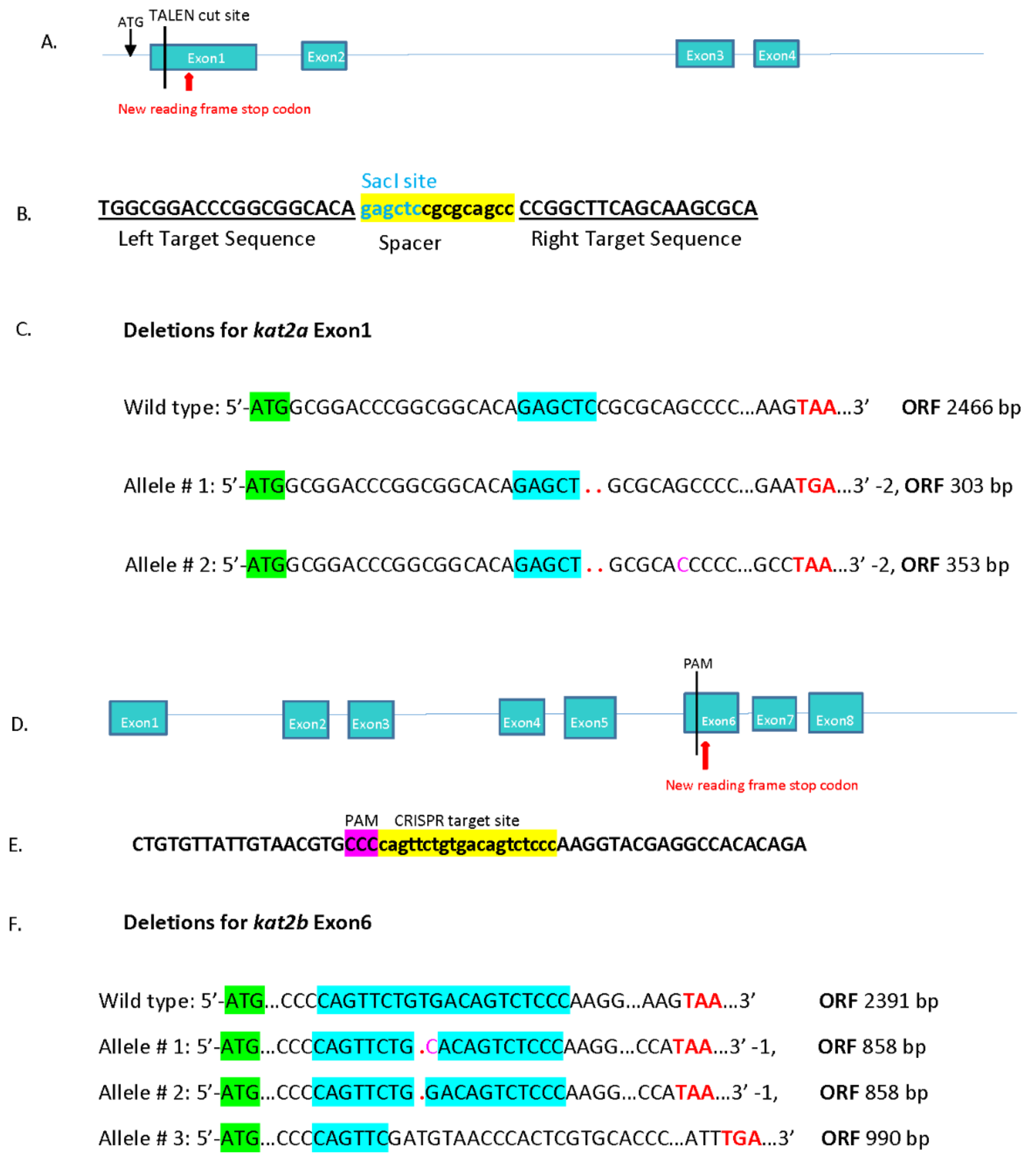

Figure S1. TALEN and CRISPR mutations created in *kat2a* and kat2b, respectively. (A) Schematic of *kat2a* locus. TALEN cut site marked with a black line, new reading frame stop codon marked with red arrow. (B) Schematic of TALEN recognition sequence for gene editing *kat2a.* Spacer sequence marked in yellow containing a SacI restriction enzyme site marked in blue. (C) Sequencing revealed deletions and mutations caused by *kat2a* TALEN. *kat2a*^{CO1007} (Allele #1) was used in all experiments reported here. (D) Schematic of *kat2b* locus. CRISPR cut site marked with a black line, new reading frame stop codon marked with red arrow. (E) Schematic of protospacer adjacent motif (PAM), marked in pink, and CRISPR recognition sequence, marked in yellow for gene editing *kat2b.* (F) Sequencing revealed deletions and mutations caused by *kat2b* CRISPR. *kat2b*^{CO1008} (Allele #1) was used in all experiments reported here.

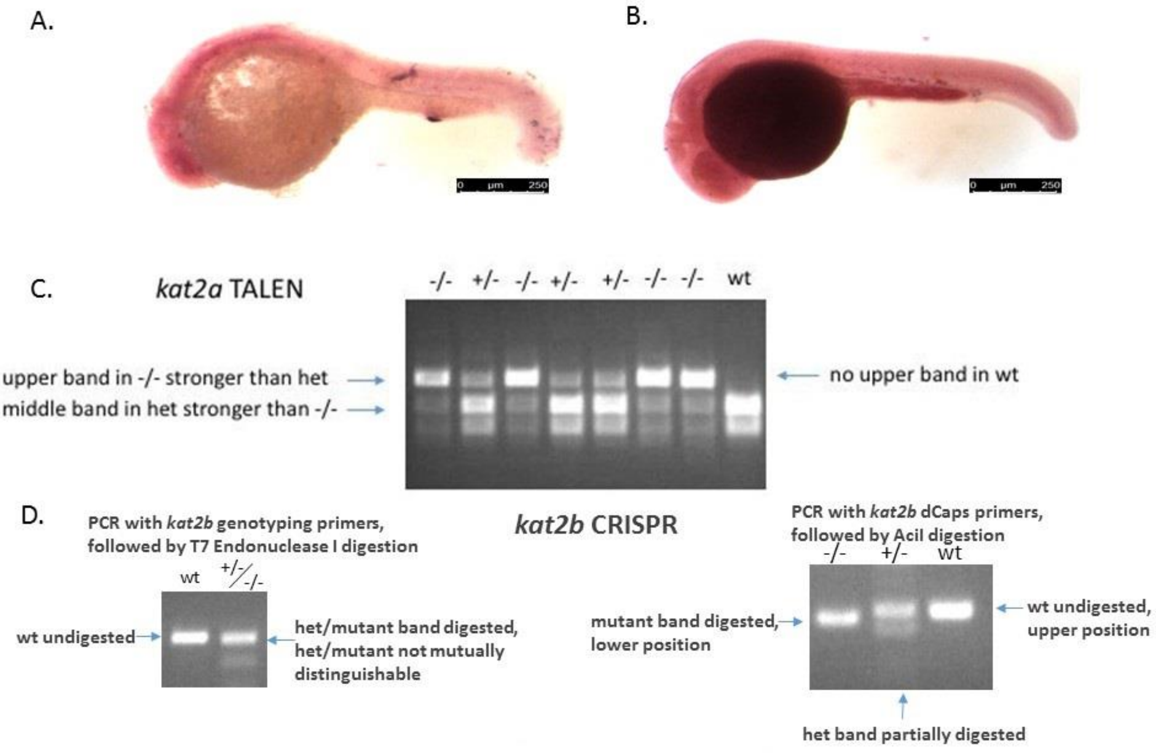

Figure S2. Expression of *kat2a* and *kat2b* in is reduced in mutants; genotyping strategy for *kat2a* and *kat2b* mutants, heterozygotes and WTs. Lateral views of *in situ* hybridization for (A) *kat2a* in *kat2a-/-* and (B) *kat2b* in *kat2b-/-* zebrafish at 36 hpf. (C) Genotyping PCR to distinguish between *kat2a* wildtype, heterozygotes (het, +/-) and mutants (-/-). PCR was performed with primers designed to amplify the TALEN target region in exon 1 of *kat2a*. PCR products were digested with SacI, an enzyme whose target sequence that lies within the TALEN site, and whose sequence is altered in the TALEN mutants. Band patterns of digested products indicate their genotype as labeled. (D) Genotyping PCR to validate mutagenesis of *kat2b* by CRISPR/Cas9. Left panel shows PCR amplification of *kat2b* exon 6 CRISPR target region using primers targeted to that region. PCR followed by T7 Endonuclease I yields the respective band patterns as labeled. Right panel shows PCR amplification of *kat2b* exon 6 CRISPR target region using a dCaps forward primer designed against the mutant sequence. Digestion of PCR product by AciI yields the respective band patterns as labeled. Scale bars represent 250 µm.

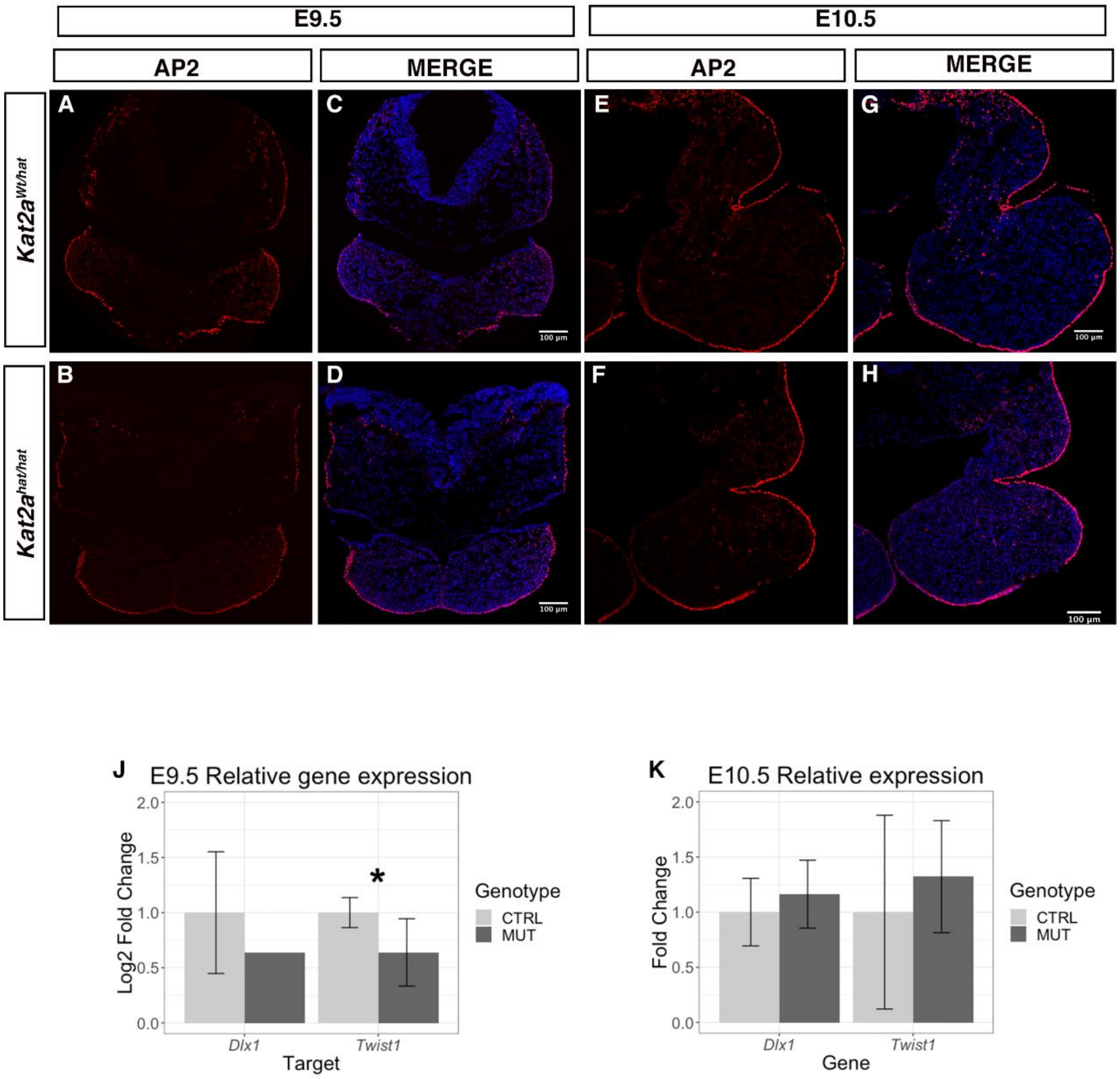

Figure S3. cNCC specification and early branchial patterning are unchanged in E9.5 and E10.5 *Kat2a*$^{hat/hat}$ mouse mutants. (A-H) Immunofluorescence with AP2 antibody and DAPI counterstain on mutant and control cryosections. (A,C) E9.5 *Kat2a*$^{Wt/hat}$ control sections and (B,D) E9.5 *Kat2a*$^{hat/hat}$ mutant sections; (C,D) merge of AP2 and DAPI. (E-H) E10.5 cryosectioned embryos. (E,G) E10.5 *Kat2a*$^{Wt/Hat}$ control sections and (F,H) E10.5 *Kat2a*$^{Hat/Hat}$ mutant sections, merge of AP2 and DAPI (G,H). (J) RT-qPCR quantification of *Dlx1* and *Twist1* relative gene expression at E9.5 using RNA extracted from the facial prominences. (K) RT-qPCR quantification of *Dlx1* and *Twist1* relative gene expression at E10.5 using RNA extracted from the facial prominences. In all cases, gene expression was normalized to GAPDH. *Kat2a*$^{Wt/hat}$ is Ctrl on graphs, *Kat2a*$^{hat/hat}$ is MUT on graphs. Y axis shows Log2 Fold change following ddCT method, gene expression was normalized to GAPDH, n = 3; * denotes statistical significance, $P<0.05$ with Welch's T-Test. Error bars show standard error of the mean. Scale bars represent 100 µm.

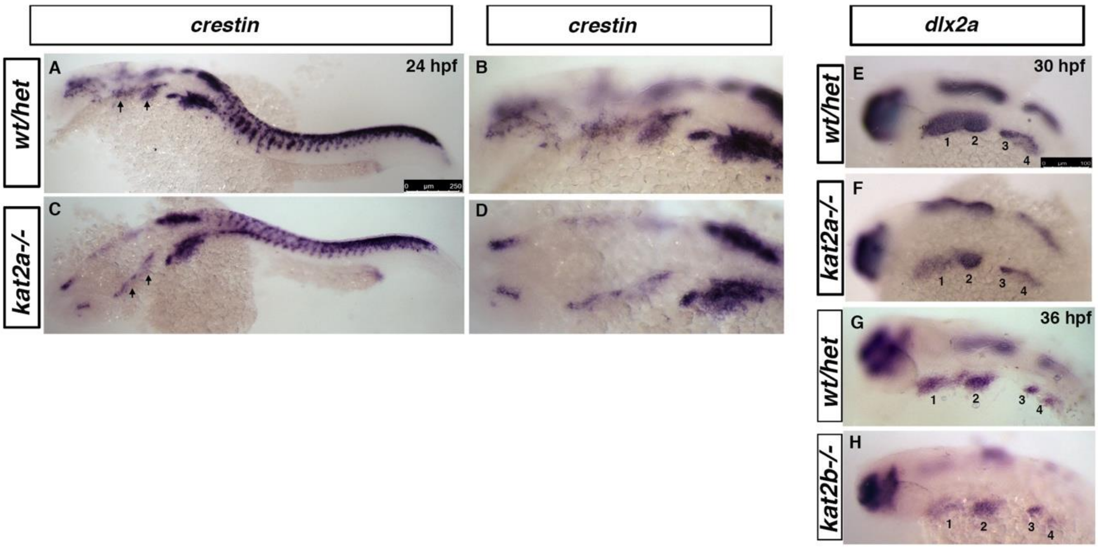

Figure S4. *crestin* and *dlx2* expression are slightly reduced in *kat2a-/-* and *kat2b-/-*. *In situ* hybridization for *crestin* (A–D) at 24 hpf, and *dlx2a* (E-H) at 30 hpf and 36 hpf as labeled. Zebrafish embryos used are wt or het (A, B, E, G), *kat2a-/-* (C, D, F), and *kat2b-/-* (H). Arrowheads (A,C) indicate pharyngeal arches, which are numbered 1-4 (E-H). Scale bars represent 250 μm (A, C) and 100 μm (E-H).

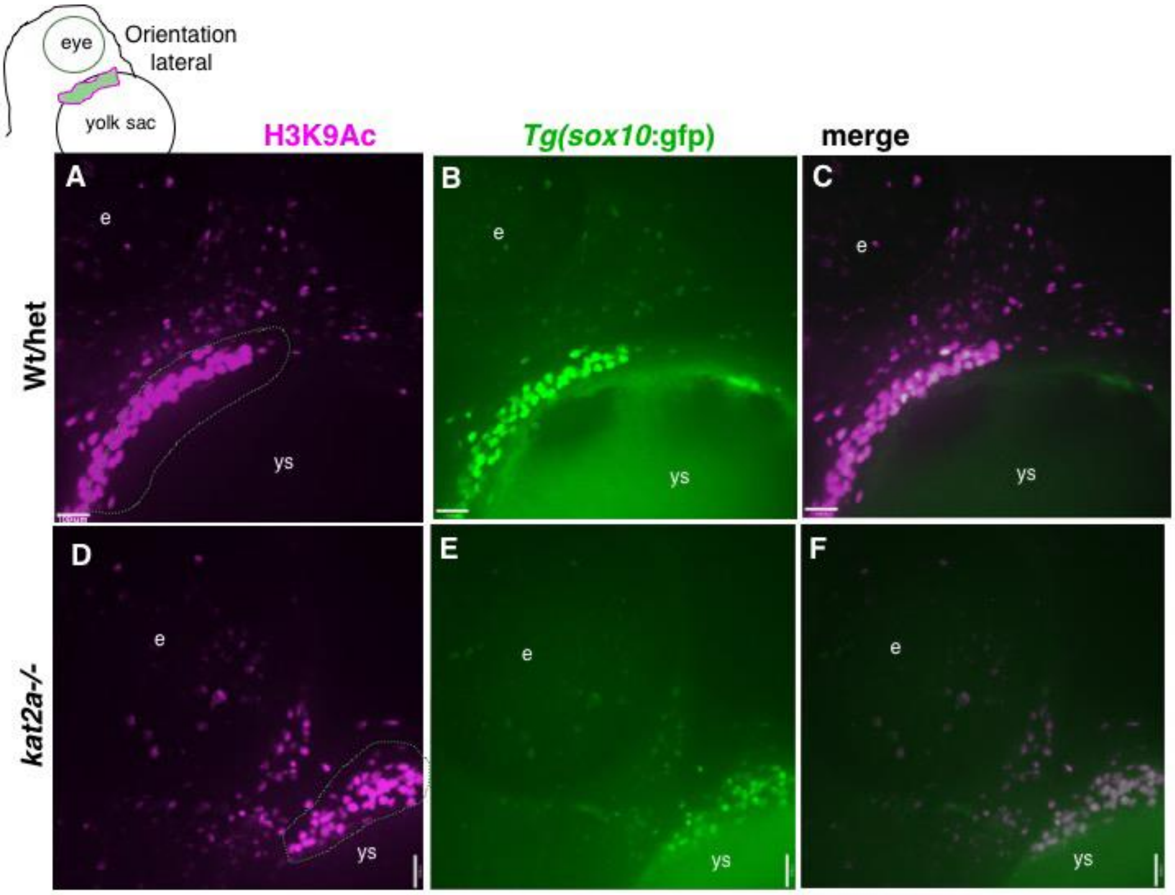

Figure S5. H3K9Ac is reduced in *kat2a-/-* mutants. Confocal micrograph for antibody staining of H3K9Ac (A, D), *sox10*:GFP (B, E) in wt (A–C) and *kat2a-/-* (D–F) whole-mount zebrafish embryos at 48 hpf, in lateral views oriented as indicated in schematic. e, eye, ys, yolk sac. Scale bars represent 100 μm.
